# Self-reported fertility impairments and help-seeking strategies among young women in Malawi

**DOI:** 10.1080/17441692.2021.1965179

**Published:** 2021-08-11

**Authors:** Jasmine Fledderjohann

**Affiliations:** Department of Sociology, Lancaster University, Lancaster, United Kingdom

**Keywords:** Infertility, Malawi, fertility, impairment, reproductive, health care, help-seeking

## Abstract

This paper analyses wave 4 the Tsogolo la Thanzi survey of *n* = 1349 Malawian women aged 16–26 to explore the prevalence and predictors of self-reported fertility impairments (difficulties conceiving and/or difficulties carrying a pregnancy to term) and help-seeking strategies. Using descriptive statistics, logistic regression models, and graphic displays, the correlates of self-reporting an impairment and patterns of help-seeking strategies are examined. Nearly 13% (*n* = 117) of those who had ever tried to conceive reported experiencing a fertility impairment. Age was positively associated with reporting an impairment, while there was a negative association with education and with parity. Of women who reported an impairment, 85.5% sought help. Visiting a hospital or clinic was the most common response, followed closely by going to a traditional healer. Around one-quarter employed multiple help-seeking strategies, highlighting the need for various help-seeking behaviours to be viewed in tandem rather than in isolation.

## Introduction

Infertility is commonly defined as the inability to conceive or maintain a pregnancy after 12+ months of regular intercourse (Gnoth et al., 2005; WHO, 2015). One-in-four couples in the Global South experience infertility (WHO, 2013), with some of the highest rates of infertility in the world found in sub-Saharan Africa (SSA) (Mascarenhas et al., 2012). Infertility can have devastating psychosocial and economic effects, including elevated levels of depression, anxiety, grief, stigmatisation, domestic violence, marital discord, poverty, lower quality of life and well-being, poorer health, and low self-esteem (Alhassan et al., 2014; Bornstein et al., 2020; Dierickx et al., 2018; Dyer, 2007; Dyer & Patel, 2012; Fledderjohann, 2012, 2017; Hollos et al., 2009; Hollos & Larsen, 2008; Naab et al., 2013; Rao et al., 2018; Rouchou, 2013; Thoma et al., 2021).

Making treatment and support available to individuals who struggle to conceive is thus vital not only for securing reproductive health, but also for improving health and well-being more broadly. Yet evidence on help-seeking strategies of individuals in SSA who self-identify as having difficulties conceiving is rare. While some research has focused on specific avenues (e.g. studies of experiences with traditional healers) for help-seeking (Barden-O’Fallon, 2005a; Parrott, 2014), these studies are exceptional. Extant research tends to be qualitative and small-scale in nature, focusing on specific help-seeking behaviours in isolation, with little known about patterns of pursuing multiple help-seeking strategies simultaneously. The availability and quality of biomedical tests and treatments for infertility has been growing in SSA, and both men and women are increasingly utilising clinical services for fertility impairments (Parrott, 2014). However, infertility remains a neglected public health issue (Asemota & Klatsky, 2015; Feldman-Savelsberg, 2002; Gipson et al., 2020; Rouchou, 2013). This paper examines the correlates of self-identified fertility impairments in Malawi, and documents the array of help-seeking strategies utilised by young women with self-identified impairments.

### Family formation Malawi

Malawi, located in Eastern Africa, is a country of about 19 million people, projected to grow to 38 million by 2050 (Population Reference Bureau, 2020). The population is very young: 44% of Malawians are under the age of 15, while only 3% are aged 65 or older. Data from 2016 show that Malawi ranks in the top ten globally for HIV infection, with an estimated prevalence of 9.2% of the adult population (US Central Intelligence Agency, 2020). Some evidence suggests uncertainty about one’s HIV sero-status may prompt desires to accelerate fertility (Trinitapoli & Yeatman, 2011).

Fertility has been falling in recent years, but is still above replacement level at 4.2 children per woman on average (Population Reference Bureau, 2020). Family formation and fertility are expected to occur early (Barden-O’Fallon, 2005b); for women, the median age at first birth is 19 years (Yeatman et al., 2019). Marriage in Malawi tends to be early and nearly universal (National Statistical Office Malawi & ICF, 2017), while divorce and remarriage are also common. Reniers (2003) found that almost half (45%) of marriages ended in divorce within 20 years. Nearly all (90%) of the women who had divorced in Reniers’s study were remarried within 10 years. Premarital fertility is low (Garenne & Zwang, 2009): Smith-Greenaway (2016) calculated that in Malawi under 3% of children were born premaritally. While sexual activity outside of marriage is common, fertility is not (Trinitapoli & Yeatman, 2011).

### Infertility in SSA

According to the WHO (2013), reproductive health ‘implies that people are able to have a responsible, satisfying and safe sex life and that they have the capability to reproduce and the freedom to decide if, when and how often to do so’. Several UN initiatives, including the Sustainable Development Goals, recognise the right to reproductive health, including control over the number and spacing of children (United Nations, 2015). By limiting one’s ability to decide if, when, and how often to reproduce, infertility comprises a pressing reproductive health problem.

Infertility is a difficult phenomenon to define and track, particularly because the scientific tools and practical goals of clinicians, public health researchers, demographers, and couples themselves may differ substantially (Fledderjohann & Barnes, 2018; Fledderjohann & Johnson, 2015; Gnoth et al., 2005; Polis et al., 2017; Thoma, 2015; Thoma et al., 2021). A key distinction is between primary infertility, defined by the WHO as the inability to become pregnant or carry a pregnancy to the point of a live birth, and secondary infertility, defined as an inability to become pregnant or carry a pregnancy subsequent to at least once previous pregnancy or live birth (WHO, 2015). Where pregnancy data may be incomplete, unreliable, or otherwise potentially biased – often a concern in prevalence studies using large-scale survey data – studies may focus on birth outcomes instead of pregnancy, identifying primary infertility as involuntary childlessness and secondary as infertility subsequent to the birth of at least one child. Although there has been a decline in infertility across the sub-continent between 1990 and 2010, estimates using population-level survey data (focusing on birth outcomes) show infertility rates in SSA are still among the highest in the world (Mascarenhas et al., 2012): As of 2010, the prevalence of primary infertility among women aged 20–44 exposed to the risk of pregnancy in SSA was 1.9% (range: 1.0%−4.0%), while secondary infertility was estimated at 11.6% (range: 3.8%–17.4%).

Importantly, these statistics rely on measures of infertility constructed from survey data, which previous research has shown may align poorly with self-identified infertility – that is, one’s own perception of their ability to conceive or produce a live birth (Fledderjohann & Johnson, 2015; Leonard, 2002). There is frequently a misalignment between clinical diagnoses, measures constructed using fertility histories in survey data, and individuals’ own perceptions of their (in)fertility (Fledderjohann & Johnson, 2015). Perceptions are highly consequential (Fledderjohann & Johnson, 2015; Leonard, 2002), as individuals act based on their own perceptions and desires, even where these do not align with external assessments. Unfortunately, large-scale survey data on self-identified infertility are difficult to come by, especially in the Global South. In a notable exception, Polis et al. (2020) found that, in a survey of ~1500 men and women in Malawi, around 8% believed it was a little or substantially likely that they were infertile or would have a difficult time becoming pregnant/impregnating a partner, with this figure climbing to as high as 20% among nulliparous women.

Childbearing is a primary goal of marriage in Malawi (Barden-O’Fallon, 2005b; Yeatman et al., 2019), and local definitions may identify a woman as infertile if she fails to conceive in as a little as a few months after marriage (Barden-O’Fallon, 2005b). Population-level survey data suggest an estimated 2% of women exposed to the risk of pregnancy experience primary infertility, and a further 10.5% experience secondary infertility (Mascarenhas et al., 2012). Recent regional data have estimated overall rates of infertility as high as 20% (Rao et al., 2018). Compared to other countries across the sub-continent, Malawi’s infertility rates place it in the upper-middle range of infertility prevalence (Barden-O’Fallon, 2005a). It remains unclear from these prevalence rates, however, how women assess their own ability to conceive and carry a pregnancy to term.

### Infertility help-seeking

Qualitative work in Malawi suggests that individuals are expected to seek help for infertility; failure to seek help is a breach of social norms (de Kok & Widdicombe, 2008). A systematic review of infertility prevalence and treatment from 2007 revealed that around just under 60% of a small sample who self-identified as infertile in rural Malawi sought treatment (Boivin et al., 2007). More recent estimates using population-based samples, however, are difficult to come by. Among individuals experiencing fertility impairments, those without access to treatment suffer greater social stigma, divorce, marginalisation, and poverty than those who are able to access care (Fledderjohann, 2012; Greil et al., 2011; Leonard, 2002; Parrott, 2014).

Despite a plethora of reproductive health programmes, limited resources have been devoted to infertility diagnosis and treatment in most countries (Dhont et al., 2011; Nachtigall, 2006). Demand for infertility services exceeds the available supply, and services are cost prohibitive for most of the world’s infertile couples (Gerrits, 2012; Mascarenhas et al., 2012); infertility care remains the preserve of wealthy couples in most countries (Asemota & Klatsky, 2015). The lack of infertility tracking and services both reflect and perpetuate social and medical systems that ignore the needs of those experiencing a fertility impairment (Barnes & Fledderjohann, 2019; Fledderjohann & Barnes, 2018; Fledderjohann & Roberts, 2018). Restricted access to health services to address infertility is a serious challenge to the tenet of reproductive justice that asserts the human right to have a child (Barnes & Fledderjohann, 2019; Fledderjohann & Barnes, 2018; Ross & Solinger, 2017). A clearer understanding of the range of help-seeking strategies infertile people utilise is a necessary step towards improving access.

### Study contributions

This paper examines self-reported fertility impairments and help-seeking strategies among young women in Malawi. I refer to ‘self-reported fertility impairments’ rather than ‘infertility’ in this paper because the analytic focus is on self-reported difficulties conceiving and/or carrying a child to term rather than clinical or demographic measures of infertility (Dick et al., 2003; Fledderjohann & Johnson, 2015; Larsen, 2000). The term ‘fertility impairments’ refers to all of the following: difficulties conceiving exclusively, difficulties carrying to term exclusively, or experiencing both difficulties. I answer two key questions: (1) What are the sociodemographic characteristics of individuals who self-identify as having a fertility impairment; and (2) among those who self-identify, who seeks treatment? I consider a range of help-seeking options, and document how treatment-seeking varies by fertility impairment.

## Methods

### Data

I accessed secondary data from Tsogolo La Thanzi (TLT)^1^, a longitudinal study of reproductive health and transitions to adulthood in an AIDS epidemic in Malawi (Trinitapoli & Yeatman, 2018; Yeatman et al., 2019). The TLT team used simple random sampling to identify a sampling frame of 15- to 25-year-olds living within 7 km of Balaka, a township in the southern region of Malawi. Respondents were asked to provide information on a wide range of topics, including reproductive health, romantic relationships, and household characteristics. Data were collected between May of 2009 and June 2012. Here, I analysed data from wave 4 of the survey, collected in June and August of 2010; this wave included questions about (possible) fertility impairments and associated help-seeking strategies.

On average, interviews took approximately 1.5 hours to complete and were conducted in Chichewa (the local language) in private rooms in a centrally located research centre (Yeatman et al., 2019). Respondents were provided with an incentive of 500 MK (~US$3.50 at the time) to compensate them for their travel expenses and time. Ethical approval for the study was provided by the Malawi National Health Sciences Research Committee (NHSRC) and by Institutional Review Boards at Arizona State University, The Pennsylvania State University, and the University of Chicago. All participants provided informed consent prior to participation. The initial sample included 1505 female respondents and featured a response rate of 96%. In wave 4, 89% of respondents from wave 1 had completed follow-up surveys, resulting in an analytic sample of *n* = 1349. Full details of the study design are available at: https://tsogololathanzi.uchicago.edu/.

### Dependent variables

**Figure 1.**
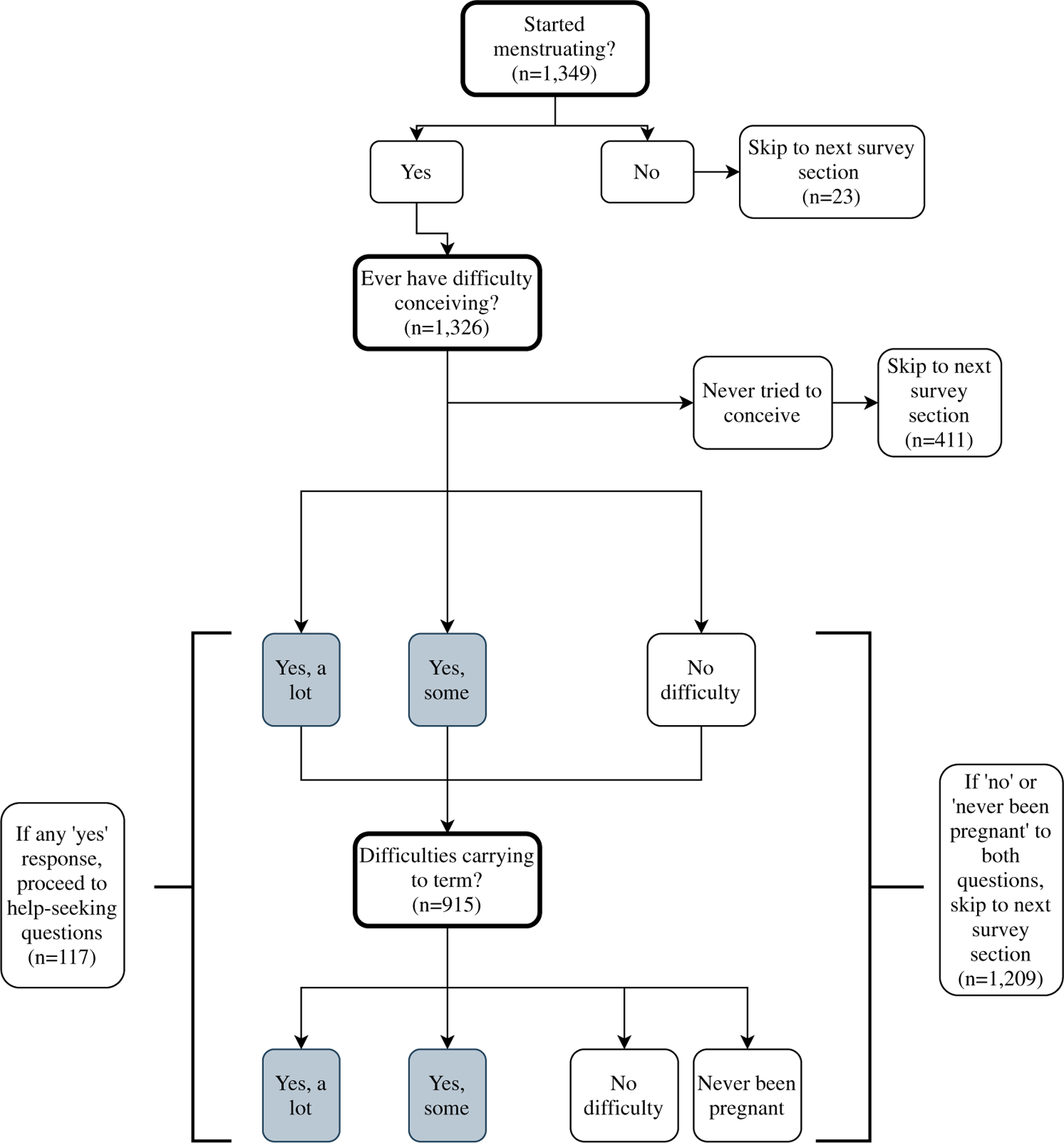
Survey skip pattern for reproductive health questions, Tsogolo La Thanzi data wave 4.

Key outcomes for this analysis were difficulties conceiving, difficulties carrying a pregnancy to term, and infertility help-seeking strategies. The measures of perceived fertility impairments came from two questions asked only in wave 4. As shown in Figure 1, respondents who indicated that they had not yet started menstruating (*n* = 23) were not asked these questions. Respondents who indicated they had begun menstruating were asked ‘Have you and a partner ever had difficulty conceiving?’ Response categories for this question included ‘yes, a lot of difficulty’, ‘yes, some difficulty’, ‘no difficulty’, and ‘never tried to conceive’. Respondents who had never tried to conceive (*n* = 411) skipped to the next survey section. I coded a dichotomous indicator for whether the respondent had ever tried to conceive.

Respondents who said they had ever tried to conceive (*n* = 915) were subsequently asked ‘Have you and a partner ever had difficulty keeping or sustaining a pregnancy up to the point of a live birth?’ Response categories were ‘yes, a lot of difficulty’, ‘yes, some difficulty’, ‘no difficulty’, and ‘never been pregnant’. Based on responses to these two questions, I generated a dichotomous measure of impaired fertility, with those who answered yes, they had a lot or yes, some difficulty conceiving and/or carrying a pregnancy to term coded 1 (*n* = 117), and those who answered no difficulty coded 0. Additionally, I coded dichotomous variables separately (i.e. disaggregated from any fertility impairment) for difficulty conceiving (*n* = 68) and difficulty carrying a pregnancy to term (*n* = 64).^2^ Respondents indicating they had never been pregnant were included in the difficulties conceiving variable, but were recoded as missing on the dichotomous indicator of difficulties carrying a pregnancy to term.

Respondents who reported a fertility impairment (*n* = 117) were also asked if they had ever engaged in any of the following help-seeking behaviours to address their fertility impairment: Going to the hospital, going to a traditional healer, finding a new partner, getting an afisi^3^, or praying/visiting a church or mosque. An open-ended ‘other’ category was also included in the survey; only one respondent reporting engaging in a help-seeking strategy was not included in the closed-ended categories. This respondent reported she ‘used a traditional drug prepared by her mother’. She had responded ‘no’ when asked if she went to a traditional healer to overcome difficulties having a child. Though her mother is presumably not a traditional healer based on these responses, I recoded her as having sought help from a traditional healer in this case, as her actions indicate using traditional medicine more broadly defined. I then coded dummy variables for each of the possible help-seeking strategies. In addition, because response categories were not mutually exclusive, I constructed dummy indicators for all possible combinations of help-seeking strategies (e.g. went to a traditional healer and prayed/visited a church or mosque, went to a hospital and a traditional healer).

### Independent variables

Building on previous evidence from SSA on important predictors of fertility behaviours, access to reproductive health care, and social pressure to conceive (Barden-O’Fallon, 2005a; Fledderjohann, 2017; Gerrits, 2012; Gibby & Luke, 2019; Grant, 2015; Grant & Furstenberg, 2007; Rouchou, 2013; Smith-Greenaway, 2016; Sundby, 2002), the key sociodemographic characteristics examined in this study include age in years, number of years of schooling completed, household wealth, total number of living children, and belief that children just happen. Due to the small sample sizes limiting statistical power, variables were coded as continuous for age, education, and parity. Household wealth is not directly measured in the TLT data. Combining the approach used in previous studies using TLT data (Oh et al., 2019; Trinitapoli & Yeatman, 2011) with a modification of the Demographic and Health Surveys approach (based on measures available in the TLT), I measured household wealth in four categories. This variable was constructed from a latent class analysis^4^ using self-reported ownership of eight different household goods (a bed with a mattress, a television, a radio, a landline or mobile telephone, a refrigerator, a bicycle, a motorcycle, and a car or truck), roofing material (coded as grass thatch, iron sheets, or asbestos/cement/other), flooring material (coded as earth/dung vs. bricks/tiles/cement/wood/other), and type of toilet (no facility, traditional pit latrine, improved pit latrine, or flush toilet), as indicators of the latent categorical variable. The respondent’s sense of human control over fertility was measured with an agree/disagree response to the statement ‘You don’t plan having children, they just happen’.

### Analytic strategy

I examined descriptive statistics for the full sample (*n* = 1349) and for two sub-samples: women who had tried to conceive (*n* = 915) and women who reported a fertility impairment (*n* = 117). I then fit logistic regression models to examine the sociodemographic correlates^5^ of self-identifying with a fertility impairment. To address the possibility of a non-random, self-selecting process of trying for a pregnancy and then self-identifying with a fertility impairment, I fit Bayesian Heckman selection models (Spiegelhalter et al., 2002), comparing results of a model assuming a selection effect to a second model fit under the assumption of no selection. Comparing deviance information criteria and log-Bayes factors for models with and without the assumption of selection, I did not find any strong evidence of selection across models. As a further robustness check, I fit multivariate multiple regression models using Stata’s *mvprobit* command, simultaneously estimating the odds of trying to conceive and odds of self-identifying with an impairment. Because the multiple regression and logistic results were extremely similar across models, I present the results from the logistic regression models for ease of interpretation. Due to small cell sizes, I focused the analysis of help-seeking on descriptive statistics only. Models were estimated using Stata v.16.

## Results

### Descriptive statistics

Table 1 displays descriptive statistics for the full sample of women, the sub-sample of women who had ever tried to conceive (and therefore were asked about perceived fertility impairments), and the sub-sample of women who self-reported an impairment (and were therefore asked about help-seeking strategies). Mean age of the full sample (*n* = 1349) was 20.59 years (range: 16–26), and just under half (49.0%) of respondents were either married or cohabiting. A further 42.6% were never married, with only a small minority being separated (0.9%), divorced (6.7%), or widowed (0.7%). Respondents in the full sample had mean of 7.42 years of education, and a somewhat low level of mean wealth (35.6% were in the lowest wealth category). In terms of fertility, 42.8% of respondents were nulliparous. Respondents had a mean of just over one living child (1.01). Around two-fifths of the sample (42.8%) believed that children just happen. Just under one-third (31%) had never tried to conceive, while 8.7% reported experiencing a fertility impairment.

The mean age in the sub-sample of women who had tried to conceive (*n* = 915) was slightly higher than in the full sample, at 21.87 years (range: 16–26). In comparison to the full sample, a substantially higher proportion of respondents who had tried to conceive were married, and far fewer were never married. The majority (71.3%) of this sub-sample were married, followed by never married (16.7%), divorced (9.6%), separated (1.3%), and widowed (1.1%). Education among this group of respondents was only slightly lower than in the full sample, with a mean value of 7.04 years of education. Women in this group were also in slightly less wealthy households (42.4% were in the lowest wealth group). Unsurprisingly given that the full sample includes women who had never tried to conceive, a smaller proportion (16.3%) of the sub-sample of women who had tried were nulliparous. Respondents in this sub-sample had a higher mean number living children (1.49) compared to the full sample. A higher percent (48.9%) of women in this sub-sample indicated a belief that children just happen.

Turning next to the sub-sample of women who self-reported an impairment (*n* = 117), they were slightly older than the previous two groups, with a mean age of 22.44 years (range: 16–26). As the minimum value indicates, there were still a small minority (*n* = 4) of respondents as young as 16 years old who self-reported a fertility impairment, representing 10.3% of the overall sample of 16-year-olds who had tried to conceive in the TLT data. While most 16-year-old respondents had not tried to conceive, a sizable minority (*n* = 39) – about 20% of the full sample of 16-year-olds – reported that they had.

Most respondents in the sub-sample were married (88.0%), with only 6.8% being never married and even smaller proportions being separated (0.9%), divorced (3.4%) or widowed (0.9%). Mean education was somewhat lower (6.66 years) among those who reported an impairment compared to the previous groups, and the maximum years of schooling in this sub-sample was 12, compared to 13 years in the other groups. Similarly, household wealth was somewhat lower in this group, with 44.4% being in the lowest wealth group. Over one-quarter (27.4%) of respondents in this sub-sample were nulliparous. Respondents had a higher mean number of living children (1.30) compared to the full sample, but fewer living children compared to the sub-sample of women who had tried to conceive. This stands to reason, as the sub-sample of women who reported an impairment excludes those who never tried to conceive, but also includes a large proportion of women who reported difficulties carrying a pregnancy. Nearly three-fifths (59.0%) of women in this sub-sample indicated a belief that children just happen.

Table 2 provides a breakdown of fertility impairments by type for the sub-sample of women who had tried to conceive. Among these respondents, 7.4% reported difficulty conceiving and 7.3% reported difficulty carrying a pregnancy to term, while 1.6% reported both difficulties. Disaggregating fertility impairments further, categories of difficulty conceiving were fairly evenly split: 3.9% of the sample reported having a lot of difficulty conceiving and an additional 3.5% reported some difficulty. By comparison, only 2.3% of the sub-sample of women who had tried to conceive reported a lot of difficulty carrying a pregnancy to term, while 5.0% reported some difficulty. Note, difficulties conceiving and carrying to a term taken together add up to more than 12.8% because they are not mutually exclusive categories – that is, a small subset of women (*n* = 15) reported difficulties both conceiving and carrying a pregnancy to term. Of those reporting an impairment, 45.3% reported difficulties conceiving only, 41.9% reported difficulties carrying a pregnancy to term only, and 12.8% reported experiencing both impairments when considered as mutually exclusive categories.

Table 2 also provides descriptive statistics for help-seeking strategies among the sub-sample of women reporting a fertility impairment. Most women (85.5%) who reported an impairment sought some kind of help; only 14.5% did nothing. The most common response was to go to a hospital or clinic, with nearly half (47.9%) of those reporting a fertility impairment having done so. A large minority (44.4%) went to a traditional healer, while no respondents reported finding a new partner or engaging an afisi. Around a quarter (24.8%) of respondents reporting a fertility impairment prayed or visited a church or mosque for help to obtain a pregnancy and/or live birth. As with fertility impairments, these percentages taken together add up to more than 100% because they are not mutually exclusive categories.

Over one-quarter (27.4%) of the subsample who reported a fertility impairment had employed multiple help-seeking strategies. Examining these as mutually exclusive categories, including the deployment of multiple strategies as separate categories, 17 respondents (14.5% of those reporting an impairment) did not seek any help for their impairment(s). Among those using single-strategy help-seeking, 35 respondents (29.9%) sought help exclusively at a hospital or clinic, while 26 (22.2%) relied exclusively on a traditional healer and only 7 (5.9%) relied exclusively on religious avenues – praying and/or seeking help at a church or mosque. In terms of multi-strategy help-seeking, 10 respondents (8.6%) visited both a hospital and traditional healer, 6 respondents (5.1%) visited a hospital and prayed/visited a church or mosque, 11 (9.4%) visited a traditional healer and prayed/visited a church or mosque, and 5 (4.3%) employed all three help-seeking strategies.

### Fertility impairments

Results of the logistic regression models predicting self-reported fertility impairments are presented in Table 3. Model 1 provides the multivariable results for whether the respondent reported any kind of fertility impairment for the sub-sample of women who had ever tried to conceive. Controlling for all else, there was a positive association between age and self-reporting an impairment (OR = 1.22; CI: 1.12–1.33), as well as a marginally significant negative association with education (OR = 0.91; CI: 0.84–0.98). There was also a strong negative association between parity and reporting an impairment (OR = 0.53; CI: 0.41–0.69).

Models disaggregated by type of impairment, with difficulties conceiving and difficulties carrying to term, are provided by Models 2 and 3 respectively. Controlling for all else Models 2 and 3 respectively show there was also a strong, positive association between age and self-reported difficulties conceiving (OR = 1.32; CI: 1.18–1.46) and carrying to term (OR = 1.16; CI: 1.04–1.30). Model 2 also indicates a negative association between difficulties conceiving and education in years (OR = 0.85; CI: 0.77–0.94), but Model 3 shows no evidence of an association for difficulties carrying to term and education. Parity was strongly and negatively associated with reporting both difficulties conceiving (OR = 0.37; CI: 0.27–0.52) and carrying to term (OR = 0.61; CI: 0.44–0.86).

### Help-Seeking

The final set of results focus on help-seeking behaviours among the analytic sub-sample (*n* = 117) of women who self-reported an impairment. Table 4 provides the breakdown of mutually exclusive help-seeking strategies for those who self-reported difficulties conceiving (*n* = 53), difficulties carrying to term (*n* = 49), and both difficulties (*n* = 15). There was a statistically significant (*χ*^2^ = 54.4; *p* < .000) difference in help-seeking strategies by type of fertility impairment.

Over one-fifth (22.6%) of respondents who reported difficulties conceiving did not take any action, compared to only 10.2% of those reporting difficulties carrying to term. All respondents who reported both difficulty conceiving and carry to term sought some form of help. Over half of respondents who reported difficulties conceiving (52.8%) or carrying to term (67.3%) used a single help-seeking strategy, compared to 46.7% of respondents who reported both difficulties. The most common single strategy for respondents reporting difficulties conceiving was use of a traditional healer (32.1%), while visiting a hospital was by far the most common strategy for difficulties carrying to term (55.1%). Respondents who reported both difficulties were roughly evenly split between using a hospital and a traditional healer, with no respondents who reported both difficulties relying exclusively on prayer.

Just under one-quarter of respondents reporting difficulties conceiving (24.5%) or carrying to term (22.4%) used multiple help-seeking strategies. By comparison, more than half (53.3%) of respondents who reported both difficulties used multiple strategies. Combinations involving a healer and prayer were the most frequent among respondents reporting difficulties conceiving, while those reporting difficulties carrying more frequently utilised strategies that included visiting a hospital – a pattern consistent with the distribution of single-strategy help-seeking described above. Respondents who reported both difficulties were fairly evenly spread across categories of multiple-strategy help-seeking.

## Discussion

This study showed that 12.8% of young women self-reported a fertility impairment, with over 10% of these experiencing both difficulties conceiving and difficulties carrying a pregnancy to term. Although age was an important predictor of fertility impairments, a small number of the youngest women in the sample reported a fertility impairment, with some women as young as age 16 reporting an impairment. These findings could reflect rates of infertility in the population and early exposure to risk factors for some women, but to a large extent may also reflect expectations for conception and pregnancy that may not align with biomedical probabilities for waiting times to conception and risk of miscarriage. This is consistent with previous literature which documents a gap between clinical, constructed, and self-reported measures of infertility (Fledderjohann & Johnson, 2015; Leonard, 2002).

A lower number of living children was associated with reporting difficulties conceiving and/or carrying to term. Having too few children is itself defined as infertility in some settings, while having many children may reduce the social pressure to conceive again (Barden-O’Fallon, 2005b; Dyer, 2007; Fledderjohann, 2012; Leonard, 2002; Rouchou, 2013). Reflecting that fecundity can change over time, in the former case, women may report (and act upon) fertility impairments even where there is no underlying subfecundity. In the latter case women may report no difficulties even where there is underlying subfecundity, as women who are no longer actively trying to conceive may not identify an underlying impairment (Greil et al., 2010). This situation speaks to the importance of perceptions in shaping (in)fertility behaviours, and highlights the value of self-report measures for understanding experiences of and responses to infertility (Barden-O’Fallon, 2005b; Fledderjohann & Johnson, 2015).

Only a small minority of women did not seek any help for their fertility impairment, while over a quarter relied on multiple help-seeking strategies. Examining help-seeking strategies in isolation (e.g. using clinic-based samples and focusing on biomedical responses) provides an incomplete picture of the range of strategies with which women who perceive an impairment may engage. Visiting a hospital or clinic and/or a traditional healer were particularly common strategies, while none of the women in the sample reported finding a new partner or taking an afisi. This could reflect shifting norms and strategies in response to infertility, but may also simply reflect the youth of the sample; an afisi may be seen as a more extreme measure to be taken after other options have been exhausted, meaning length of time both experiencing fertility impairments and seeking help could be important factors to model as the cohort ages.

Interestingly, when help-seeking was disaggregated, there was some evidence of differences in help-seeking by type of fertility impairment. In particular, a higher proportion of women reporting difficulties conceiving took no action compared to those reporting difficulties carrying to term, and no one who reported both difficulties did nothing. This could reflect differences in the exigency of addressing these impairments: Although not all miscarriages require medical care (and miscarriages very early in a pregnancy may go undetected), many pregnancy complications can necessitate immediate medical care, which may prompt urgent help-seeking. Conversely, there is less likely to be a medical emergency that would require the same kind of urgent engagement with help-seeking in the case of difficulties conceiving. This observation may likewise account for the fact that a higher proportion of women who reported difficulties conceiving used a traditional healer, while a higher proportion of women who reported difficulties carrying visited a hospital or clinic.

That different help-seeking strategies were associated with difficulties conceiving versus carrying may also help to explain why over half of women who experienced both difficulties utilised multiple help-seeking strategies, compared to only around a quarter of women who experienced a single type of impairment. For example, a woman who experiences both impairments may visit a traditional healer for difficulties conceiving and a hospital for difficulties carrying to term, resulting in multiple help-seeking strategies as she engages with the strategy most common to each type of impairment over time. It is also possible that women who experience both impairments are more motivated by that experience to seek help through as many avenues as possible.

There are several limitations to this study. First, the subsample of women self-identifying as having a fertility impairment is small, limiting statistical power. Second, while the TLT is rare in its inclusion of measures of women’s own perceptions of their ability to conceive and carry a pregnancy, these data are now somewhat dated. While there is no strong reason to expect that rates of perceived fertility impairments may have shifted substantially across the last decade, it is possible that help-seeking strategies may have changed both in terms of overall uptake and specific combinations of help-seeking, reflecting the growing (though still insufficient) availability of biomedical treatment options (Parrott, 2014; Thoma et al., 2021). Moreover, as the COVID-19 pandemic continues to unfold, it is not clear whether/how the pandemic is impacting the availability of different help-seeking options. This is an important area for future research.

Third, it was not possible to assess the timing of impairments nor of help-seeking, as these items were only included in wave 4 of the TLT data, and the survey contains no questions about the timing of impairments. This gives rise to several limitations. Because the questions ask whether respondents have ‘ever’ had a difficulty, some fertility impairments in the data may have been resolved, while others may be ongoing impairments. For example, 19 respondents who were currently pregnant reported a fertility impairment, with eight of these women reporting difficulties conceiving and six reporting both kinds of impairment. Whether this reflects that these were difficult pregnancies to conceive, that reported difficulties conceiving pre-dated the current pregnancy, or some other factor is unclear. It is also possible that self-perceptions of fertility impairments may change over time, both as women continue to (not) become pregnant and give birth across the reproductive lifecourse, and in retrospect as women reflect on previous experiences. Further research in future is needed to understand the timing of fertility impairments and how timing shapes women’s self-perceptions across the lifecourse. Similarly, some women may currently be seeking help, while others may have engaged in a strategy previously but have since ceased seeking help. It is not possible from these data to make causal claims as a result (nor is that the aim here). It is worth noting, however, de Kok and Widdicombe’s (2008) work on infertility in Malawi showing that cessation of help-seeking in the case of a suspected fertility impairment violates social norms, and may have substantial social consequences. Help-seeking may often not entail one discrete event, and further work on the timing of help-seeking (and its cessation) is needed.

Also linked to the issue of the unknown timing of fertility impairments, it is not possible here to assess whether or how use of hormonal or long acting reversible contraceptives (LARC) may impact women’s perceptions of fertility impairments because current contraceptive use does not necessarily reflect contraceptive use during nor prior to reported fertility impairments. Extant research has shown that some women worry that contraceptive use can lead to infertility (Boivin et al., 2020; Gueye et al., 2015; Hindin et al., 2014; Sedlander et al., 2018), which could feasibly increase reporting of perceived fertility impairments, particularly where hormonal contraceptives are associated with a delay before fertility returns. This issue represents another important area for future research.

Finally, there may be social desirability bias in reporting on help-seeking: some behaviours (e.g. seeking an afisi) in response to difficulties conceiving may be more socially acceptable than others, and there may be a conservative bias in the estimates of some behaviours. Social desirability bias may likewise apply to reporting self-identified infertility, as infertility can mark a considerable threat to adult status and adherence to gender norms (de Kok & Widdicombe, 2008; Dyer, 2007; Fledderjohann, 2012, 2017; Rouchou, 2013; Thoma et al., 2021). This points to the potential for a conservative bias in these findings, as some respondents may suspect reproductive failure (and may even have sought advice and/or treatment) but will not be willing report a fertility impairment in a survey (Fledderjohann & Johnson, 2015; Greil, 1991). On the other hand, self-identification may overestimate subfecundity, particularly where women experience impatience to conceive – that is, the propensity to self-identify before clinical definitions of infertility would suggest the need to pursue fertility testing and treatment (Leridon, 1992).

Also notably, surveys may underestimate pregnancy wastage by as much as 50% (Casterline, 1989). In part, this figure reflects the fact that early miscarriages may be missed by respondents themselves. In this case, women may misreport difficulty carrying a pregnancy to term as difficulty conceiving or as no impairment. While fertility impairments were examined as a broad category before being disaggregated by kind of impairment here, it is possible that some misclassification of impairments may occur. However, fecundity may be best understood as a spectrum rather than a dichotomous state, and the classification of underlying impairments is always prone to measurement error (Fledderjohann & Johnson, 2015; Leridon, 1992). Moreover, the perception of a fertility impairment is likely to be more salient for social outcomes (e.g. help-seeking) than is underlying subfecundity (Fledderjohann & Johnson, 2015), and so women’s own reports of their experiences are highly consequential even when misaligned with underlying biological states.

The strengths of this study lie in its use of a population-based sample to examine perceived fertility impairments and help-seeking. Much of the extant research on infertility in Malawi (and in SSA more broadly) focuses on healthcare facility-based samples and/or on measures of infertility constructed from fertility histories. This study therefore fills a gap in the literature by focusing on women’s own perceptions of fertility impairments and including women who may not access clinical spaces. Overall, the results highlight that perceptions about fertility impairments are strongly associated with fertility histories, and speak to the value of using self-report measures to study infertility. Results also indicate that use of multiple help-seeking strategies is common, indicating that further research is needed to understand how and when different fertility impairments and help-seeking strategies intersect over the reproductive lifecourse. The inclusion of self-report measures of infertility and help-seeking strategies in large-scale fertility surveys is essential to situate infertility and fertility together to better-understand the reproductive health needs of individuals across the lifecourse.

## Figures and Tables

**Table 1.** Descriptive statistics, Tsogolo La Thanzi wave 4.

	Full Sample (*n* = 1349)				Tried to Conceive (*n* = 915)					Self-reported an Impairment (*n* = 117)					
Variable name	Mean/Percent	Std. Dev.	Min	Max	Mean/Percent	Std. Dev.	Min	Max	Difference from Full Sample	Mean/Percent	Std. Dev.	Min	Max	Difference from Full Sample	Difference from Tried to Conceive
Age (years)	20.59	3.30	16	26	21.87	2.92	16	26	*X*^2^ = 481.9; *p* < .001	22.44	2.68	16	26	*X*^2^ = 57.6; *p* < .001	*X*^2^ = 17.5; *p* = .064
Marital Status															
Married/Cohabiting	49.0%	0.50	0	1	71.3%	0.45	0	1	*X*^2^ = 563.8; *p* < .001	88.0%	0.33	0	1	*X*^2^ = 78.1; *p* < .001	*X*^2^ = 18.4; *p* < .001
Separated	0.9%	0.09	0	1	1.3%	0.11	0	1	*X*^2^ = 5.7; *p* = .017	0.9%	0.09	0	1	*X*^2^ = 0.0; *p* = .966	*X*^2^ = 0.2; *p* = .642
Divorced	6.7%	0.25	0	1	9.6%	0.29	0	1	*X*^2^ = 37.3; *p* < .001	3.4%	0.18	0	1	*X*^2^ = 2.3; *p* = .133	*X*^2^ = 5.9; *p* = .015
Widowed	0.7%	0.09	0	1	1.1%	0.10	0	1	*X*^2^ = 4.8; *p* = .029	0.9%	0.09	0	1	*X*^2^ = 0.0; *p* = .881	*X*^2^ = 0.1; *p* = .791
Never Married	42.6%	0.49	0	1	16.7%	0.37	0	1	*X*^2^ = 780.3; *p* < .001	6.8%	0.25	0	1	*X*^2^ = 67.1; *p* < .001	*X*^2^ = 9.4; *p* = .002
Highest education completed (years)	7.42	2.77	0	13	7.04	2.81	0	13	*X*^2^ = 111.1; *p* < .001	6.66	2.85	0	12	*X*^2^ = 15.1; *p* = .300	*X*^2^ = 6.4; *p* = .932
Household wealth															
Lowest	35.6%	0.48	0	1	42.2%	0.49	0	1	*X*^2^ = 54.1; *p* < .001	44.4%	0.50	0	1	*X*^2^ = 4.4; *p* = .036	*X*^2^ = 0.3; *p* = .596
Lower-Middle	20.3%	0.40	0	1	22.5%	0.42	0	1	*X*^2^ = 8.5; *p* = .004	22.2%	0.42	0	1	*X*^2^ = 0.3; *p* = .591	*X*^2^ = 0.0; *p* = .936
Higher-Middle	23.1%	0.42	0	1	20.2%	0.40	0	1	*X*^2^ = 13.5; *p* < .001	16.2%	0.37	0	1	*X*^2^ = 3.4; *p* = .064	*X*^2^ = 1.3; *p* = .251
Highest	21.0%	0.41	0	1	15.1%	0.36	0	1	*X*^2^ = 59.7; *p* < .001	17.1%	0.38	0	1	*X*^2^ = 1.2; *p* = .280	*X*^2^ = 0.4; *p* = .515
Nulliparous	42.8%	0.49	0	1	16.3%	0.37	0	1	*X*^2^ = 815.2; *p* < .001	27.4%	0.45	0	1	*X*^2^ = 12.4; *p* < .001	*X*^2^ = 12.1; *p* = .001
Parity	1.01	1.08	0	5	1.49	1.00	0	5	*X*^2^ = 815.4; *p* < .001	1.30	1.07	0	4	*X*^2^ = 12.8; *p* = .025	*X*^2^ = 12.3; *p* = .031
Believes children just happen	42.8%	0.50	0	1	48.9%	0.50	0	1	*X*^2^ = 41.9; *p* < .001	59.0%	0.49	0	1	*X*^2^ = 13.6; *p* < .001	*X*^2^ = 5.5; *p* = .019
Self-reported impairment	8.7%	0.28	0	1	12.8%	0.33	0	1	*X*^2^ = 60.8; *p* < .001						
Reported difficulties conceiving	5.0%	0.22	0	1	7.4%	0.26	0	1	*X*^2^ = 34.0; *p* < .001						
Reported difficulties carrying to term	4.7%	0.21	0	1	7.0%	0.26	0	1	*X*^2^ = 31.9; *p* < .001						
Sought some form of help	7.4%	0.26	0	1	10.9%	0.31	0	1	*X*^2^ = 51.2; *p* < .001						
Never tried to conceive	31.0%	0.46	0	1											

**Table 2.** Descriptive statistics for fertility impairments and help-seeking, Tsogolo La Thanzi wave 4.

Variable name	Percent
Self-identified fertility impairment (*n* = 915)	12.8%
Experienced difficulty conceiving (*n* = 915)	7.4%
A lot of difficulty	3.9%
Some difficulty	3.5%
No difficulty	92.6%
Experienced difficulty carrying to term (*n* = 875)	7.3%
A lot of difficulty	2.3%
Some difficulty	5.0%
No difficulty	92.7%
Experienced both difficulties (*n* = 915)	1.6%
Sought help for fertility impairment (*n* = 117)	85.5%
Did nothing	14.5%
Went to hospital	47.9%
Went to traditional healer	44.4%
Found a new partner	0.0%
Took an Afisi	0.0%
Prayed/visited church or mosque	24.8%

**Table 3.** Logistic regression results for models predicting fertility impairments, Tsogolo La Thanzi wave 4

	Model 1		Model 2		Model 3	
	Any difficulties		Difficulties conceiving		Difficulties carrying to term	
	OR	CI	OR	CI	OR	CI
Age (years)	1.22***	[1.12,1.33]	1.32***	[1.18,1.46]	1.16**	[1.04,1.30]
Highest education completed (years)	0.91*	[0.84,0.98]	0.85**	[0.77,0.94]	0.94	[0.85,1.05]
Household wealth						
Lowest	0.82	[0.44,1.52]	0.71	[0.31,1.62]	0.75	[0.34,1.65]
Lower-Middle	0.79	[0.41,1.52]	0.88	[0.38,2.04]	0.82	[0.37,1.83]
Higher-Middle	0.65	[0.33,1.30]	0.83	[0.35,1.99]	0.44	[0.17,1.12]
Highest (ref)						
Parity	0.53***	[0.41,0.69]	0.37***	[0.27,0.52]	0.61**	[0.44,0.86]
Believes children just happen	1.49	[0.98,2.27]	1.64	[0.95,2.83]	1.20	[0.70,2.04]
Observations	915		915		875	

Notes:

* *p*<.05

** *p*<.01

*** *p*<.001;

Sample size of *n* = 875 in Model 3 reflects a legitimate skip, where some of the 915 respondents who had tried to conceive had never become pregnant, and so were not asked about difficulties carrying to term.

**Table 4.** Mutually exclusive categories of help-seeking by type of fertility impairment, Tsogolo La Thanzi wave 4.

Help Sought	Difficulties Conceiving	Difficulties Carrying	Both Difficulties	Total
Hospital	5	27	3	35
	9.4%	55.1%	20.0%	29.9%
Traditional Healer	17	5	4	26
	32.1%	10.2%	26.7%	22.2%
Prayed	6	1	0	7
	11.3%	2.0%	0.0%	6.0%
Hospital and Healer	4	4	2	10
	7.6%	8.2%	13.3%	8.6%
Hospital and Prayed	0	5	1	6
	0.0%	10.2%	6.7%	5.1%
Healer and Prayed	8	1	2	11
	15.1%	2.0%	13.3%	9.4%
Hospital, Healer, and Prayed	1	1	3	5
	1.9%	2.0%	20.0%	4.3%
Did Nothing	12	5	0	17
	22.6%	10.2%	0.0%	14.5%
Total	53	49	15	117
	100%	100%	100%	100%

Notes: Frequencies and percents given; *χ*^2^ = 54.4; *p* < .000

## Data Availability

The data that support the findings of this study are openly available in the University of Michigan ICPSR data bank at https://doi.org/10.3886/ICPSR36863.v3, reference number ICPSR 36863.

## Footnotes

1. The TLT data were initially funded for 8 waves of data collection; these waves formed the first phase of data collection, TLT-1, analysed here. Additional funding was secured for subsequent phases of data collection, but these data are not yet publicly available for download.

2. Note, the number of respondents reporting difficulties conceiving and difficulties carrying to term add up to more than the n=117 women reporting a fertility impairment because n=15 women reported both difficulties, and so are coded 1 on both variables.

3. A term referring to taking a partner outside of the relationship to conceive. This option is distinguished from taking a new partner in that an afisi is a sexual relationship taken in secret for the purpose of conceiving, and the child is likely to be passed off as the romantic partner’s child.

4. I compared model fit statistics and distribution of marginal probabilities across categories for 3, 4, and 5 category versions of a wealth variable, with the 4-category variable being the best fit.

5. Although marital status is a key correlate of self-identification, I was not able to include marital status in these models because nearly all women who reported an impairment were married, leading to unstable models. Nearly 90% of women reporting a fertility impairment in this analytic sub-sample were married. Nonetheless, I included marital status in reporting descriptive statistics, as marital status is a tremendously important predictor of fertility behaviors in Malawi, and it varied substantially between the full sample and analytic sub-samples (further details in Table 1 in the Results section).
